# Comparative effects on glucose absorption of intragastric and post-pyloric nutrient delivery in the critically ill

**DOI:** 10.1186/cc11522

**Published:** 2012-09-17

**Authors:** Anna E Di Bartolomeo, Marianne J Chapman, Antony V Zaknic, Matthew J Summers, Karen L Jones, Nam Q Nguyen, Christopher K Rayner, Michael Horowitz, Adam M Deane

**Affiliations:** 1 Discipline of Acute Care Medicine, University of Adelaide, North Terrace, Adelaide, SA 5000, Australia; 2 National Health and Medical Research Council of Australia, Centre for Clinical Research Excellence in Nutritional Physiology and Outcomes, Level 6, Eleanor Harrald Building, North Terrace, Adelaide, SA 5000, Australia; 3 Intensive Care Unit, Level 4, Emergency Services Building, Royal Adelaide Hospital, North Terrace, Adelaide, SA 5000, Australia; 4 Discipline of Medicine, University of Adelaide, Royal Adelaide Hospital, Level 6, Eleanor Harrald Building, North Terrace, Adelaide, SA 5000, Australia; 5 Department of Gastroenterology, Level 4, Emergency Services Building, Royal Adelaide Hospital, North Terrace, Adelaide, SA 5000, Australia

## Abstract

**Introduction:**

Studies in the critically ill that evaluate intragastric and post-pyloric delivery of nutrient have yielded conflicting data. A limitation of these studies is that the influence in the route of feeding on glucose absorption and glycaemia has not been determined.

**Methods:**

In 68 mechanically ventilated critically ill patients, liquid nutrient (100 ml; 1 kcal/ml containing 3 g of 3-O-Methyl-D-glucopyranose (3-OMG), as a marker of glucose absorption), was infused into either the stomach (n = 24) or small intestine (n = 44) over six minutes. Blood glucose and serum 3-OMG concentrations were measured at regular intervals for 240 minutes and the area under the curves (AUCs) calculated for 'early' (AUC_60_) and 'overall' (AUC_240_) time periods. Data are presented as mean (95% confidence intervals).

**Results:**

Glucose absorption was initially more rapid following post-pyloric, when compared with intragastric, feeding (3-OMG AUC_60_: intragastric 7.3 (4.3, 10.2) vs. post-pyloric 12.5 (10.1, 14.8) mmol/l.min; *P *= 0.008); however, 'overall' glucose absorption was similar (AUC_240_: 49.1 (34.8, 63.5) vs. 56.6 (48.9, 64.3) mmol/l.min; *P *= 0.31). Post-pyloric administration of nutrients was also associated with greater increases in blood glucose concentrations in the 'early' period (AUC_60_: 472 (425, 519) vs. 534 (501, 569) mmol/l.min; *P *= 0.03), but 'overall' glycaemia was also similar (AUC_240_: 1,875 (1,674, 2,075) vs. 1,898 (1,755, 2,041) mmol/l.min; *P *= 0.85).

**Conclusions:**

In the critically ill, glucose absorption was similar whether nutrient was administered via a gastric or post-pyloric catheter. These data may have implications for the perceived benefit of post-pyloric feeding on nutritional outcomes and warrant further investigation.

## Introduction

Marked malnutrition in the critically ill is associated with increased morbidity [1,2]. While feeding by the nasogastric route is preferred, approximately 50% of critically ill patients fail to meet their caloric needs using this approach [3,4]. When this occurs, nutrient is frequently delivered directly into the small intestine, bypassing the stomach [5], in the belief that this will increase caloric delivery and thereby optimise nutritional therapy, leading to improved outcomes [6]. However, data that have evaluated caloric intake during either intragastric or post-pyloric delivery are inconsistent, which may relate to the time taken in placing post-pyloric feeding [7-11]. Moreover, the premise that small intestinal feeding will increase absorption has not been tested.

We have reported that glucose absorption is markedly impaired in the critically ill, both following gastric and small intestinal nutrient administration [12,13]. Glucose is not absorbed within the stomach, and so absorption will be limited both by the rate of gastric emptying. However, we recently observed that even when administered directly into the small intestine, glucose absorption is markedly diminished in critical illness [12]. This latter finding leads to the hypotheses that factors distal to the pylorus impair glucose absorption in the critically ill, so that post-pyloric nutrient delivery may not increase nutrient absorption when compared to intragastric delivery. If post-pyloric feeding does not increase nutrient absorption, it is unlikely to improve clinical outcomes.

Hyperglycaemia occurs frequently in critically ill patients and is detrimental to patient outcomes [14]. Furthermore, variability in glycaemia may be just as, or even more, harmful than elevated mean glucose concentrations [15]. Given that the rate of nutrient delivery from the stomach into the small intestine is a major determinant of 'postprandial' glycaemic excursions in the critically ill, as well as in health and diabetes [12,16,17], it is important to determine whether the route of enteral feeding influences glycaemia in this group.

The primary aim of this study was to evaluate the effect of the route of enteral feeding (intragastric or post-pyloric) on glucose absorption in the critically ill, with the secondary aim of determining effects on glycaemia.

## Materials and methods

We undertook a retrospective analysis of data collected over a 42-month period (September 2006 to March 2010) in 68 mechanically ventilated, critically ill patients admitted to a mixed surgical/medical Intensive Care Unit. These patients were enrolled in studies where glucose absorption was measured after intragastric or post-pyloric nutrient administration using identical study protocols [12,13,18].

### Subjects

All patients were either receiving, or suitable to receive, enteral nutrition via intragastric or small intestinal catheters. Exclusion criteria included: pregnancy; contraindication to enteral nutrient or requirement for supplemental parenteral nutrition; previous surgery on the oesophagus, stomach or duodenum; gastrointestinal surgery during that same hospital admission; and a history of diabetes mellitus. In patients receiving intragastric feeds insulin was administered if blood glucose concentrations were >15 mmol/l [12], whereas during post-pyloric nutrient delivery insulin was administered as per unit protocol (>10 mmol/l) [13]. Prokinetic drugs were withheld for 24 hours prior to study in both groups. All studies were approved by the Royal Adelaide Hospital Human Ethics Committee and performed in accordance with local legal requirements for research conducted on unconscious patients. Written, informed consent was obtained from the next of kin.

### Protocol

All patients were fasted for at least six hours prior to commencement of the study [13]. In the intragastric feeding group a nasogastric feeding tube was inserted approximately 50 cm below the nares, according to standard practice guidelines [19]. The intragastric position of the tube was confirmed using abdominal radiography and pH testing of aspirates [20]. Small intestinal feeding catheters were inserted using electromagnetic (n = 32) or endoscopic techniques (n = 12) [21]. Post-pyloric placement was confirmed using either abdominal radiograph, scintigraphy and/or transmucosal potential difference [22]. After the position of the feeding tube was confirmed, the study 'meal' was infused via the feeding tube over six minutes. This consisted of 100 mL of Ensure^® ^(Abbott Laboratories, Bedford, MA, USA; 1 kcal/ml, 64% carbohydrate), mixed with 3 g of 3-O-Methyl-D-glucopyranose (3-OMG) (Sigma-Aldrich, Castle Hill, NSW, Australia) dissolved in 5 ml of water. t_0 _was considered to be the time that the infusion of the meal was completed. Arterial blood samples were taken immediately prior to nutrient infusion (t = -6) and at regular timed intervals (t = 5, 15, 30, 45, 60, 75, 90, 120, 150, 180, 210 and 240 minutes) for the measurement of 3-OMG and blood glucose concentrations.

### Measurements

#### Blood glucose and 3-OMG concentrations

Arterial blood glucose concentrations were determined using a portable glucose meter (Medisense Precision QID, Abbott Laboratories). Glucometers were calibrated prior to each study. Glucose absorption was estimated using serum 3-OMG concentrations [12,13]. Blood (5 ml) was collected at regular intervals (t = -6 to 240 minutes) with serum being separated by centrifugation (3,200 rpm for 15 minutes at 4°C) and stored at -70°C for subsequent analysis using High Performance Liquid Chromatography (HPLC) [13].

#### Statistical analysis

Data are reported as mean (95% confidence interval), and presented in the figures as mean (standard deviation (SD)), unless stated otherwise. Summary data (that is, t0-60 and t0-240) were generally peaked and, therefore, areas under the concentration curve (AUC), calculated using the trapezoidal rule, were used as measures. Data were assessed for normality and lack of heteroscedalascity and these assumptions were met in all cases.

Analyses of 'early' and 'overall' time points, that is, t60 and t240 minutes were chosen a priori [13]. The rate of gastric emptying was anticipated to markedly affect absorption, particularly in the 'early' time period (AUC60), but to have more modest influence on 'overall' absorption (AUC240). Total glucose absorption reflects the extent of substrate absorbed over that time period as indicated by the area under the serum 3-OMG concentration curve (AUC), whereas the rate of absorption influences the time taken to reach the peak serum 3-OMG concentrations, while the magnitude of the peak reflects both of these factors [23]. Independent sample t-tests were used for analyses and significance was defined as P <0.05. An independent biostatistician had access to all data and used SPSS 18 (SPSS Inc., Chicago, Illinois, United States of America) for analyses.

Relationships were assessed using Pearson Correlation and evaluated between (i) 'initial' glucose absorption (3-OMG AUC_60_) and changes in blood glucose concentration at t_60_; and (ii) peak 3-OMG concentrations and the maximum increment in blood glucose concentration [13].

## Results

Twenty-four patients were recruited in studies where they were fed via the intragastric route and 44 patients received post-pyloric feeding. There were no significant differences in age, weight or body mass index, Acute Physiology and Chronic Health Evaluation (APACHE) II scores, serum creatinine or administration of sedative and analgesic drugs between the two groups (Table 1). Patients in the small intestinal feeding group were studied later in their admission to the Intensive Care Unit.

**Table 1:** Demographic data

Feeding site	Intragastric	Post-pyloric
**Subjects (n) **	24	44
**Age (years)**	52 (45, 60)	52 (47, 57)
**Sex (n) Male**	19 (79%)	36 (82%)
**Body Mass Index (kg/m^2^)**	26 (23, 28)	27 (25, 29)
**APACHE II Score:**		
**Admission †**	21 (11 to 38)	20 (7 to 40)
**Day of Study†**	16 (6 to 27)	16 (4 to 30)
**Admission diagnosis**		
**Trauma**	6 (25%)	13 (30%)
**Pneumonia/Respiratory**	5 (21%)	13 (30%)
**Sepsis (Other source)**	4 (17%)	5 (12%)
**Neurosurgical/Neurology**	4 (17%)	3 (7%)
**Burn injury**	2 (8%)	3 (7%) 3 (7%)
**Cardiac surgery/**	2 (8%)	
**Cardiology**		4 (9%)
**Pancreatitis**	1 (4%)	
**Time from admission to study day (days) †**	4 (1 to 10)	8 (1 to 38)*
**Insulin administered during study (number (%))**	0 (0%)	20 (45%) **
**Sedative and analgesic drugs administration^1 ^(number (%))**	16 (67%)	21 (48%)

**Serum creatinine (μmol/l)**	**68 (19 to 248)**	**66 (29 to 357) **

**^1 ^**Sedative and analgesic drug administration was defined as fentanyl or propofol on study day. (APACHE II, Acute Physiology and Chronic Health Evaluation II) Data are mean (95% confidence intervals) or † median (range). * denotes *P *= 0.004 **P <0.001

### Blood glucose concentrations

**Figure 1:**
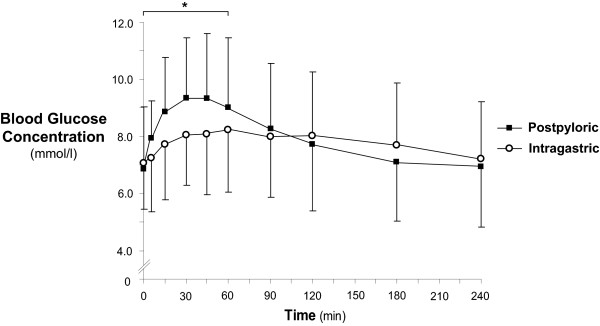
**Blood glucose concentrations following administration of nutrient via intragastric and post-pyloric routes**. For the initial 60 minutes after the infusion, glycaemic excursions were greater following post-pyloric administration of nutrient (*P = 0.03), but, over the entire 240 minutes, glucose concentrations were comparable between the two groups (*P *= 0.85). Data are mean ± SD.

Fasting blood glucose concentrations were comparable between the groups (intragastric 7.1 (6.3, 7.9) vs. post-pyloric 6.9 (6.4, 7.3) mmol/l; *P *= 0.58) (Figure 1). In both groups, blood glucose rose after nutrient administration (*P* <0.001). Despite insulin use, post-pyloric delivery of nutrient was associated with greater glycaemic excursions in the 'early' period (AUC_60_: intragastric 472 (425, 519) vs. post-pyloric 534 (501, 569) mmol/l.min; *P *= 0.03), and there was a trend for an increase in the peak excursion (9.2 (8.4, 10.1) vs. 10.2 (9.5, 10.8) mmol/l; *P *= 0.09). Blood glucose concentrations at t_60 _(8.2 (7.3, 9.2) vs. 9.0 (8.3, 9.8) mmol/l; *P *= 0.19) and 'overall' glycaemia (AUC_240_: 1,875 (1,674, 2,075) vs. 1,898 (1,755, 2,041) mmol/l.min; *P *= 0.85) were similar in the two groups.

### Serum 3-OMG concentrations (glucose absorption)

**Figure 2:**
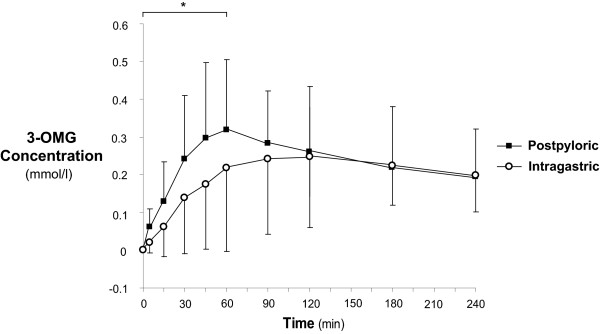
**Glucose (3-OMG) absorption following administration of nutrient via intragastric and post-pyloric routes**. 'Early' glucose absorption was increased following post-pyloric delivery (* *P *= 0.008). While 'overall' glucose absorption was similar between intragastric and post-pyloric feeding route (*P *= 0.31). Data are mean ± SD.

In all patients, 3-OMG concentrations increased after both infusions, and at study end (t_240_), remained greater than zero in all patients (Figure 2). However, administration of nutrient directly into the small intestine resulted in increased glucose absorption during the 'early' period when compared with intragastric feeding (AUC_60 _intragastric 7.3 (4.3, 10.2) vs. post-pyloric 12.5 (10.1, 14.8) mmol/l.min; *P *= 0.008). Small intestinal feeding was also associated with a reduced time to peak (Time to Peak 3-OMG: 132 (100, 164) vs. 78 (61, 95) minutes; *P *= 0.001), although there was no difference in 3-OMG peak concentrations in the two groups (0.29 (0.20 to 0.39) vs. 0.37 (0.31 to 0.43) mmol/l; *P *= 0.13). 'Overall' glucose absorption (AUC_240_) was similar between intragastric and post-pyloric feeding route (AUC_240 _49.1 (34.8, 63.5) vs. 56.6 (48.9, 64.3) mmol/l.min; *P *= 0.31).

### Relationships between 3-OMG and blood glucose concentrations

In the whole group there was a relationship between rise in glycaemia and glucose absorption (3-OMG AUC_60 _and Δ blood glucose concentration at t_60 _when compared to fasting glucose, r = 0.50; *P* <0.001, and serum 3-OMG and blood glucose concentrations at t_60 _r = 0.41; *P* <0.001). There was also an association between the maximum increment in blood glucose and the rate of glucose absorption (for example, 3-OMG peak and Δmax in blood glucose, r = 0.37; *P *= 0.02).

## Discussion

The key observation in this study is that small intestinal delivery of nutrient, when compared to intragastric administration, appeared to have little effect on 'overall' glucose absorption over 240 minutes.

To our knowledge, this study is the first to compare the effect of route of enteral feeding (gastric versus small intestinal) on glucose absorption in the critically ill. Previous studies have quantified glucose absorption following gastric and small intestinal administration independently [12,13]. Glucose absorption following intragastric administration is known to be markedly impaired in critical illness compared to 'health', and the slower initial rate of absorption is, at least in part, attributable to delayed gastric emptying [12]. However, glucose absorption following small intestinal administration is also attenuated in the critically ill, indicating that small intestinal factors are pivotal to the malabsorption of glucose in this group [13]. While the underlying mechanisms are as yet unknown, several 'small intestinal' abnormalities that have the capacity to reduce glucose absorption have been observed in the critically ill, including abnormal small intestinal motility [6], and mucosal abnormalities, such as decreased villus height and crypt depth [24]. The effects of critical illness on the microstructure of the small intestine in humans are unknown, but in an animal model of critical illness a reduction in expression of the duodenal sodium-glucose co-transporter was evident suggesting defective glucose transportation by enterocytes [25].

Some authorities recommend that enteral nutrition be preferentially delivered via small intestinal feeding catheters [26]. The proposed benefits of this approach are an increase in nutrient delivery and a reduction in nosocomial pneumonia [9,27,28]. However, our study suggests that glucose absorption, particularly after the 'initial' period, may be similar, regardless of the route of delivery. Because of this observation the perceived nutritional benefits of post-pyloric feeding are open to reevaluation. While small intestinal feeding may potentially reduce the risk of nosocomial pneumonia by a reduction in gastric volumes and, thereby, gastro-oesophageal reflux and pulmonary aspiration [27], the latter outcomes were not assessed in our study. However, the relationship between gastroesophageal reflux and slow gastric emptying has not been established in the critically ill [29].

Marked hyperglycemia leads to adverse outcomes in the critically ill [30]. In addition, glycaemic variability (or wider swings in glycaemia) appears to be an independent predictor of mortality [15]. The rate of gastric emptying influences, and is influenced by, blood glucose concentrations in 'health', type-2 diabetes and critical illness [12,17,31]. Our study suggests that delivery of glucose directly into the small intestine, which bypasses the regulatory effect of gastric emptying, has the potential to exacerbate glycaemic excursions. It is, therefore, plausible that any potential benefits from post-pyloric nutrition may be negated by inferior glycaemic control. Future studies that evaluate the effects of route of feeding (that is, intragastric vs. post-pyloric) should consider the potential impact on glycaemia.

There are limitations to this study that should be recognised. The route of feeding was not randomised, but, despite this, the groups appeared to be well matched according to most parameters, including illness severity. The post-pyloric feeding cohort were studied later in their ICU stay, and abnormalities within the gastrointestinal tract have been reported to improve during a patient's admission [32]. This would, if anything, have favoured improved glucose absorption in this group, which was not observed. Insulin protocols varied due to the retrospective nature of the study. The insulin regimen was more intensive in the patients fed by the post-pyloric route with insulin administered at lower blood glucose concentrations. Possibly due to both a more intensive insulin regimen and/or higher blood glucose concentrations, more patients in the post-pyloric cohort received insulin, but given that insulin *per se*, may increase small intestinal glucose absorption [33], the similar glucose absorption observed in the two routes of feeding is even more remarkable. It should be recognised that it cannot be assumed that the increase in glycaemic excursions was exclusively due to post-pyloric delivery of nutrient, as there may have been other factors that influenced glucose metabolism (for example, counter-regulatory hormones including glucagon) that were not measured.

Due to the retrospective design, our study may have been underpowered. Sixty-eight subjects, however, represents a substantial cohort, and this sample was sufficient to show a mean difference in AUC_240 _3-OMG concentrations (glucose absorption) of 4.0 mmol/l.min, with β and α errors of 0.8 and 0.05 respectively. Differences less than this are unlikely to be of clinical significance. Another potential limitation is that the meal was administered as a bolus, which may have implications for the interpretation of our data. In principle, glucose absorption in the post-pyloric group may have been underestimated, because delivery of nutrient into the small intestine at rates that are above those of 'normal' gastric emptying could, conceivably, lead to suboptimal absorption. On the other hand, slow gastric emptying in the intragastric feeding cohort would have introduced a bias towards reduced absorption in that group, rather than showing comparable effects to post-pyloric feeding. It is also possible that delivery of a bolus into the small intestine exacerbated glycaemic excursions, because the rate of entry of glucose into the small intestine is a major determinant of glycaemia in health, type-2 diabetes and critical illness [12,17,31].

Despite these limitations, our observations highlight the need to undertake prospective studies to confirm and/or further explore our hypothesis-generating results. In particular, a prospective randomised study evaluating intragastric versus small intestinal feeding at infusions rates that better reflect routine practice would be desirable.

## Conclusions

Small intestinal administration of nutrient increases the initial, but does not appear to affect the 'overall', rate of glucose absorption, when compared to gastric administration. However, more rapid initial glucose absorption during post-pyloric feeding has the potential to affect glycaemia adversely. Further studies examining the impact of the route of feeding on nutrient absorption and nutritional outcomes are desirable.

## Key messages

• When compared to intragastric administration, post-pyloric delivery of nutrient did not increase the glucose absorption over a four-hour period.

• The perceived benefit of post-pyloric feeding on nutritional outcomes warrants further evaluation.

## Abbreviations

3-OMG: 3-O-Methyl-D-glucopyranose; APACHE II: Acute Physiological and Chronic Health Evaluations II; AUC: area under the curve; HPLC: high performance liquid chromatography

## Competing interests

The authors declare that they have no competing interests.

## Authors' contributions

ADiB was the main contributor to study design, the acquisition, analysis and interpretation of the data, and drafting of the manuscript. MJC, KLJ, NQN, CKR and MH all contributed to study conception and revision of the manuscript. AVZ and MJS were responsible for data acquisition and analysis and contributed to revision of the manuscript. AMD supervised ADiB and participated in drafting the manuscript. All authors read and approved the final manuscript.
